# An immunohistochemical study of the granulysin expression of 6 types of proven adverse cutaneous drug reaction

**DOI:** 10.1186/2045-7022-4-S3-P3

**Published:** 2014-07-18

**Authors:** Marie Weinborn, Annick Barbaud, François Truchetet, Philippe Beurey, Lucie Germain, Jean-Luc Schmutz, Bernard Cribier

**Affiliations:** 1 Nancy University Hospital, Dermatology Department, France; 2 Metz Thionville Hospital, Dermatology Department, France; 3 Pathology Center Emile Gallé, Pathology Center, France; 4 Nancy University Hospital, Clinical Epidemiology and Evaluation Department, France; 5 Strasbourg University Hospital, Dermatology Department, France

## Background

In Steven Johnson's syndrome (SJS) and Toxic epidermal necrolysis (TEN), Chung et al. demonstrated that granulysin is the key cytotoxic molecule. But the specificity of granulysin in SJS-TEN is actually discussed. We studied the granulysin expression of 6 types of cutaneous adverse drug reaction (CADR) with a proven diagnosis (maculopapular exanthema (MPE), Drug Reaction with Eosinophilia and Systemic Symptoms (DRESS), fixed drug eruption (FDE), SJS, TEN, acute generalized exanthematous pustulosis (AGEP)).

## Method

This retrospective histological analysis consisting of second reading of anonymous slides. It was about skin biopsies (B) from patients during a proven CADR (chronological investigation, single attributable drug or/and skin tests realised). We used an immunohistochemical (IC) technique (clone RF10, MBL DA86-3) to study the granulysin expression. The distribution pattern of granulysin was analysed qualitatively (localisation) and graded semiquantitatively as absent, mild, moderate or severe (0 to 3).

## Results

102 biopsies were analysed resulting from 98 patients. For the 4 additional biopsies, twice were realised during the same episode. The discharge diagnosis was MPE in 31 patients (34 B), DRESS in 24 (25 B), SJS in 14, TEN in 7, FDE in 10 (11 B) and AGEP in 12. Among these 102 prelevements, the IC technique didn't work in 3 cases. The skin tests were done in 77%, for the remaining 23% a single drug was attributable. We observed that the granulysin was expressed in these 6 CADR with different intensities and localisations. There was no statistically difference between SJS and NET. In MPE the granulysin is expressed in the epidermis but only mildly in 41%. In DRESS cases, the expression is more intense and frequent (76%) (p=0.0002). In the dermis, in MPE and DRESS there is the same number of positively slides (88%). In MPE the expression is mild and principaly superficial, whereas in DRESS it is mostly moderate or intense and often deeper with statistically significative differences. Between DRESS and SJS+NET there are few statistically differences, except in the superficial dermis the expression is more intense in DRESS cases (p=0.0071).

## Conclusion

This study allowed to determine that the granulysin expression is not specific to SJS and TEN and bring to the fore that it is also strongly expressed in the DRESS. The IC expression of the granulysin could help to distinguish MPE to the DRESS particularly in the early phase.

